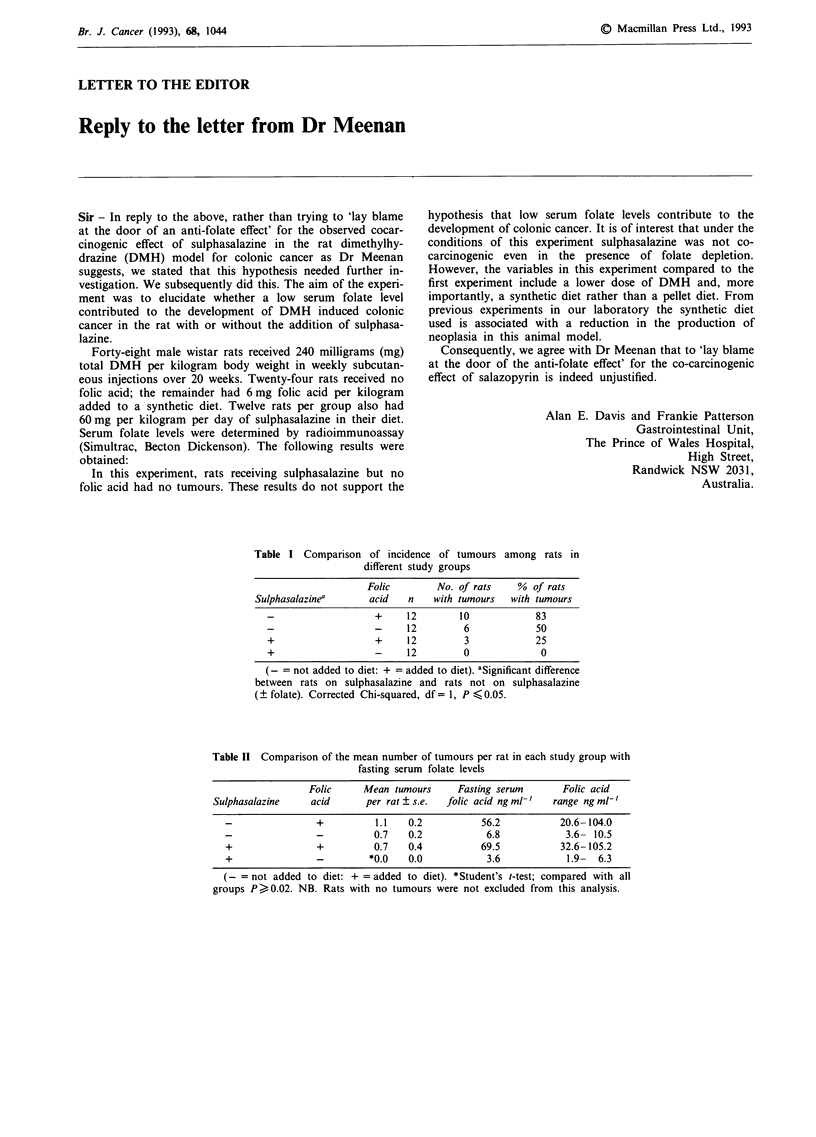# Reply to the letter from Dr Meenan

**Published:** 1993-11

**Authors:** Alan E. Davis, Frankie Patterson


*Br. J. Cancer* (1993), **68**, 1044 © Macmillan Press Ltd., 1993

LETTER TO THE EDITOR

### Reply to the letter from Dr Meenan

**Sir** – In reply to the above, rather than trying to 'lay blame at the door of an anti-folate effect' for the observed cocarcinogenic effect of sulphasalazine in the rat dimethylhydrazine (DMH) model for colonic cancer as Dr Meenan suggests, we stated that this hypothesis needed further investigation. We subsequently did this. The aim of the experiment was to elucidate whether a low serum folate level contributed to the development of DMH induced colonic cancer in the rat with or without the addition of sulphasalazine.

Forty-eight male wistar rats received 240 milligrams (mg) total DMH per kilogram body weight in weekly subcutaneous injections over 20 weeks. Twenty-four rats received no folic acid; the remainder had 6 mg folic acid per kilogram added to a synthetic diet. Twelve rats per group also had 60 mg per kilogram per day of sulphasalazine in their diet. Serum folate levels were determined by radioimmunoassay (Simultrac, Becton Dickenson). The following results were obtained:

In this experiment, rats receiving sulphasalazine but no folic acid had no tumours. These results do not support the hypothesis that low serum folate levels contribute to the development of colonic cancer. It is of interest that under the conditions of this experiment sulphasalazine was not co-carcinogenic even in the presence of folate depletion. However, the variables in this experiment compared to the first experiment include a lower dose of DMH and, more importantly, a synthetic diet rather than a pellet diet. From previous experiments in our laboratory the synthetic diet used is associated with a reduction in the production of neoplasia in this animal model.

Consequently, we agree with Dr Meenan that to 'lay blame at the door of the anti-folate effect' for the co-carcinogenic effect of salazopyrin is indeed unjustified.

Alan E. Davis and Frankie Patterson
Gastrointestinal Unit,
The Prince of Wales Hospital,
High Street,
Randwick NSW 2031,
Australia.

**Table I** Comparison of incidence of tumours among rats in different study groups

| *Sulphasalazine*[a] | *Folic acid* | *n* | *No. of rats with tumours* | *% of rats with tumours* |
|---|---|---|---|---|
| – | + | 12 | 10 | 83 |
| – | – | 12 | 6 | 50 |
| + | + | 12 | 3 | 25 |
| + | – | 12 | 0 | 0 |

(– = not added to diet: + = added to diet). [a]Significant difference between rats on sulphasalazine and rats not on sulphasalazine (± folate). Corrected Chi-squared, df = 1, $P \leqslant 0.05$.

**Table II** Comparison of the mean number of tumours per rat in each study group with fasting serum folate levels

| *Sulphasalazine* | *Folic acid* | *Mean tumours per rat ± s.e.* | | *Fasting serum folic acid ng ml$^{-1}$* | *Folic acid range ng ml$^{-1}$* |
|---|---|---|---|---|---|
| – | + | 1.1 | 0.2 | 56.2 | 20.6–104.0 |
| – | – | 0.7 | 0.2 | 6.8 | 3.6– 10.5 |
| + | + | 0.7 | 0.4 | 69.5 | 32.6–105.2 |
| + | – | *0.0 | 0.0 | 3.6 | 1.9– 6.3 |

(– = not added to diet: + = added to diet). *Student's *t*-test; compared with all groups $P \geqslant 0.02$. NB. Rats with no tumours were not excluded from this analysis.